# Primary monophasic synovial sarcoma of the cervical esophagus confirmed by detection of the SS18-SSX2 fusion transcripts: case report and literature review

**DOI:** 10.1186/s40792-020-00940-8

**Published:** 2020-07-20

**Authors:** Ken Sasaki, Masahiro Noda, Yusuke Tsuruda, Yasuto Uchikado, Itaru Omoto, Yoshiaki Kita, Takaaki Arigami, Shinichiro Mori, Hiroshi Kurahara, Akihiro Nakajo, Michiyo Higashi, Takao Ohtsuka

**Affiliations:** 1 Department of Digestive Surgery, Breast and Thyroid Surgery, Graduate School of Medical and Dental Sciences, Kagoshima University, 8-35-1 Sakuragaoka, Kagoshima-shi, Kagoshima, 890-8520 Japan; 2 Department of Onco-Biological Surgery, Graduate School of Medical and Dental Sciences, Kagoshima University, 8-35-1 Sakuragaoka, Kagoshima-shi, Kagoshima, 890-8520 Japan; 3 Department of Pathology, Graduate School of Medical and Dental Sciences, Kagoshima University, 8-35-1 Sakuragaoka, Kagoshima-shi, Kagoshima, 890-8520 Japan

**Keywords:** Synovial sarcoma, Esophagus, SS18-SSX2, Monophasic

## Abstract

**Background:**

Synovial sarcoma (SS) of the esophagus is extremely rare. Because of the microscopic features of SS, the monophasic type can easily be misdiagnosed as other spindle cell tumors. Here, we present the first case of a primary SS of the esophagus in the presence of SS18-SSX2 fusion transcripts.

**Case presentation:**

A 47-year-old Japanese woman was initially diagnosed with thyroid papillary carcinoma in the left lobe and leiomyoma of the cervical esophagus and subsequently underwent left thyroid lobectomy and enucleation of the esophageal tumor. Four years after the first surgery, the esophageal tumor recurred. Endoscopic biopsy of the tumor revealed atypical cell proliferation with spindle cell features and mitoses. Immunohistochemistry showed focal positivity for bcl-2 and HHF35. Furthermore, the presence of *SS18*-*SSX*2 fusion transcripts was confirmed by reverse transcription-polymerase chain reaction analysis, using a paraffin-embedded tumor specimen. Therefore, the tumor was diagnosed as monophasic SS of the cervical esophagus. We re-evaluated the surgical specimen enucleated 3 years previously, which was initially diagnosed as leiomyoma, and the diagnosis of SS was confirmed. The patient underwent cervical esophagectomy with isolated jejunal interposition reconstruction. Three years after the second surgery, SS recurred in the distal anastomotic site between the jejunum and the esophagus, and the patient underwent thoracoscopic esophagectomy with gastric conduit reconstruction. The pathological grade of the lesion worsened with every recurrence.

**Conclusions:**

Monophasic SS can be difficult to discriminate from other spindle cell tumors based on microscopy alone, and molecular analysis could be useful for confirming the precise diagnosis of monophasic SS.

## Background

Synovial sarcoma (SS) is a soft tissue malignancy typically occurring in close proximity to the large joints of the extremities in young adults and with a male preponderance [1]. No cellular origin has yet been proven; however, current research suggests that it might develop from primitive mesenchymal cells or myoblasts [2, 3]. SS does not arise from the synovium although named so because of its microscopic resemblance to normal synovium. SS of the gastrointestinal tract, including the esophagus, is extremely rare. The monophasic fibrous type can easily be misdiagnosed as other spindle cell tumors because of the microscopic features of SS. Immunohistochemistry (IHC) is routinely used in the differential diagnosis; however, there is no specific marker for SS. The majority of patients with SS carry the pathognomonic t(X;18) (p11.2;q11.2) translocation, resulting in fusion of the SS18 (formerly SYT) gene on chromosome 18 with an *SSX* gene on chromosome X [4]. Here, we report the first case of monophasic SS of the esophagus in the presence of *SS18*-*SSX2* fusion transcripts detected by reverse transcription-polymerase chain reaction (RT-PCR) analysis using a paraffin-embedded tumor specimen, which was originally misdiagnosed as leiomyoma.

## Case presentation

**Fig. 1:**
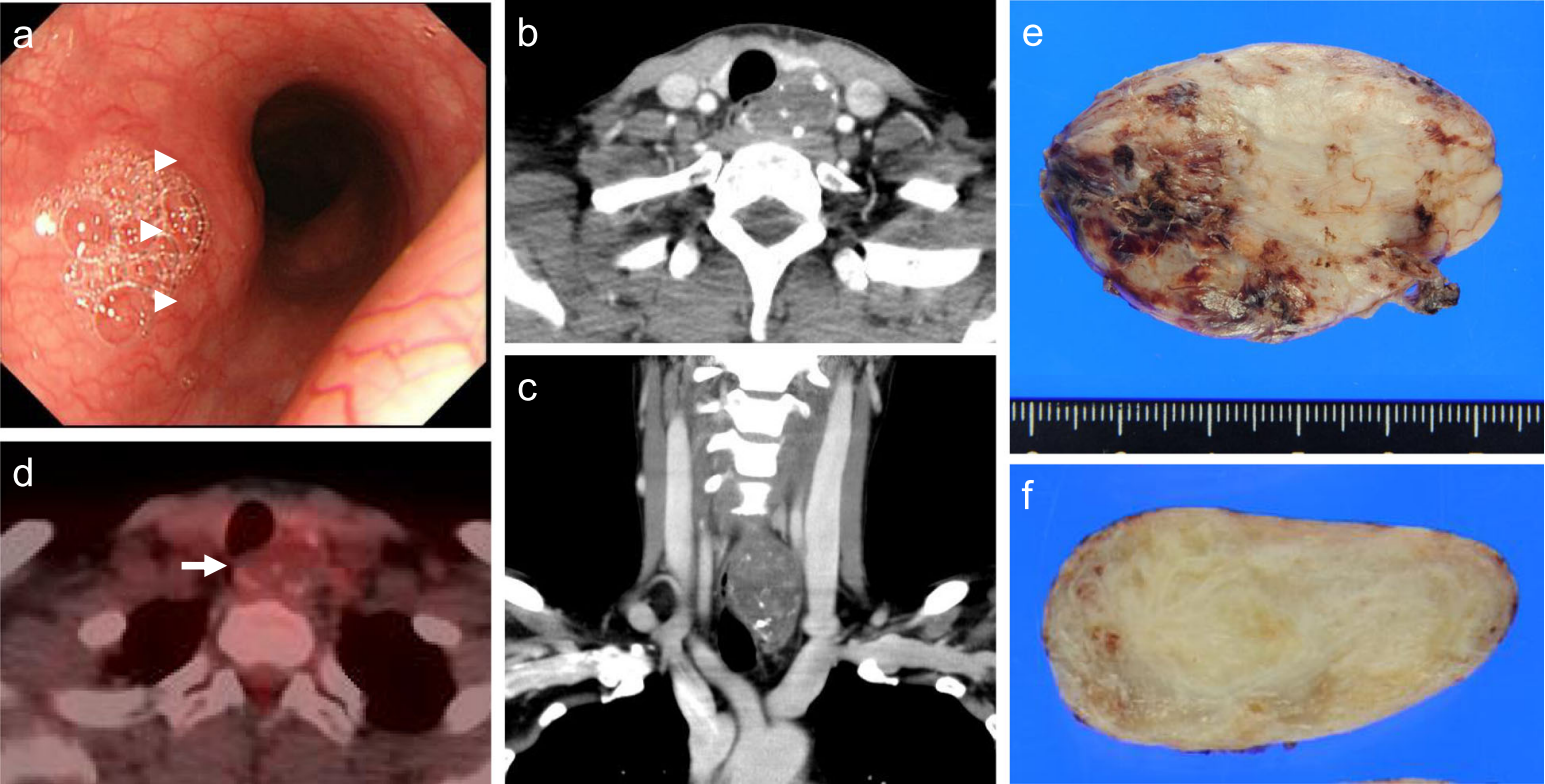
Images obtained before the initial surgery. Esophagoscopy showed a 50-mm-sized submucosal tumor (arrowheads) (**a**). Contrast-enhanced computed tomography (CT) showed an oval-shaped mass with limited calcification in the cervical esophagus (**b**, **c**). ^18^F-fluorodeoxyglucose positron-emission tomography/CT (FDG-PET/CT) showed no FDG uptake in the esophageal tumor (arrow) (**d**). Macroscopic findings of the initial tumor. It was oval and smooth (**e**), and its incised surface was yellow–white, homogeneous, and elastic hard (**f**)

A 47-year-old Japanese woman was referred to our hospital because of thyroid papillary carcinoma in the left lobe and esophageal submucosal tumor (SMT). All her laboratory data were within the normal ranges. Esophagoscopy showed a 50-mm-sized tumor, 18 cm from the incisor, covered with intact mucosa and located at the left wall of the cervical esophagus (Fig. 1a). A contrast-enhanced computed tomography (CT) revealed an oval-shaped mass with limited calcification, measuring 52 × 34 × 21 mm in the cervical esophagus (Fig. 1b, c), whereas neither lymph node swelling nor distant metastasis was observed. ^18^F-fluorodeoxyglucose positron-emission tomography/computed tomography (FDG-PET/CT) did not reveal any FDG uptake in the esophageal lesion (Fig. 1d). The histological diagnosis based on percutaneous ultrasound-guided core needle biopsy analysis was spindle cell tumor. IHC revealed negative staining for c-kit, CD34, desmin, HHF35, SMA, and S-100, and the MIB-1 index was < 1%. The patient was initially diagnosed with thyroid papillary carcinoma and leiomyoma of the cervical esophagus and underwent left thyroid lobectomy and enucleation of the esophageal tumor. Macroscopically, the tumor was oval and smooth (Fig. 1e), and its incised surface was yellow–white, homogeneous, and elastic hard (Fig. 1f). Histological examination of the surgical specimen of the esophagus reestablished the diagnosis of leiomyoma.

**Fig. 2:**
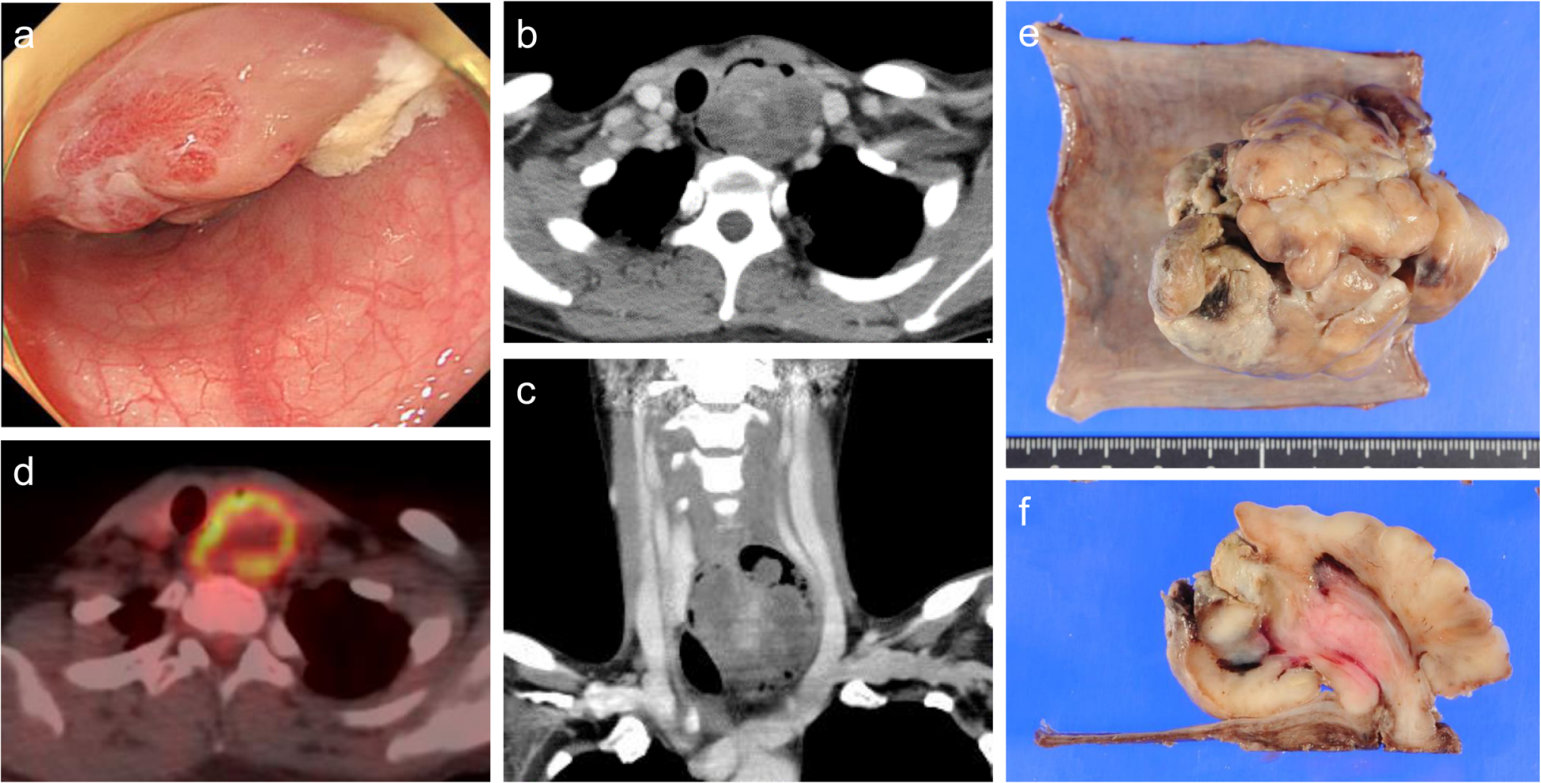
Images obtained before the second surgery. Esophagoscopy showed a 70-mm-sized protruding tumor (**a**). Contrast-enhanced CT showed a well-circumscribed mass in the cervical esophagus (**b**, **c**). FDG-PET/CT showed FDG uptake in the esophageal tumor (**d**). Macroscopic findings of the secondary tumor. It was pedunculated and multilobulated and covered by the thinning esophageal mucosa (**e**), while its incised surface was whitish tan and had areas of focal hemorrhage (**f**)

Four years after the first surgery, the cervical esophageal tumor recurred. Esophagoscopy showed a 70-mm-sized protruding tumor located at the left wall of the cervical esophagus 18 cm from the incisor (Fig. 2a). A contrast-enhanced CT revealed a well-circumscribed mass in the cervical esophagus (Fig. 2b, c). FDG-PET/CT showed FDG uptake in the tumor (Fig. 2d). Microscopy of the endoscopic biopsy revealed atypical cell proliferation in the lesion with spindle cell features and the presence of a few mitoses. IHC showed focal positivity for bcl-2 and HHF35 and negativity for CD34, c-kit, desmin, SMA, S-100, and DOG-1. We suspected that the tumor, which was previously diagnosed as leiomyoma, was in fact SS. Therefore, we sought to confirm the presence of the SS18-SSX fusion transcript by RT-PCR using primers targeting the *SS18*, *SSX1*, *SSX2*, and *SSX4* genes, and *SS18*-*SSX2* fusion transcripts were detected. Based on these analyses, the tumor was re-diagnosed as monophasic SS of the cervical esophagus. We re-evaluated the surgical specimen enucleated 3 years previously, which was initially diagnosed as leiomyoma, and corrected the diagnosis to SS. The patient underwent cervical esophagectomy with isolated jejunal interposition reconstruction. The tumor did not invade beyond the wall from the surface of the adventitia of the esophagus, and no lymph node swelling was observed macroscopically during the operation. Macroscopically, the secondary tumor was pedunculated and multilobulated and covered by the thinning esophageal mucosa (Fig. 2e), while the incised surface was whitish tan and had areas of focal hemorrhage (Fig. 2f). Complete resection was achieved based on the pathological examination. Although adjuvant therapy was considered, it was not provided after consultation with the patient.

**Fig. 3:**
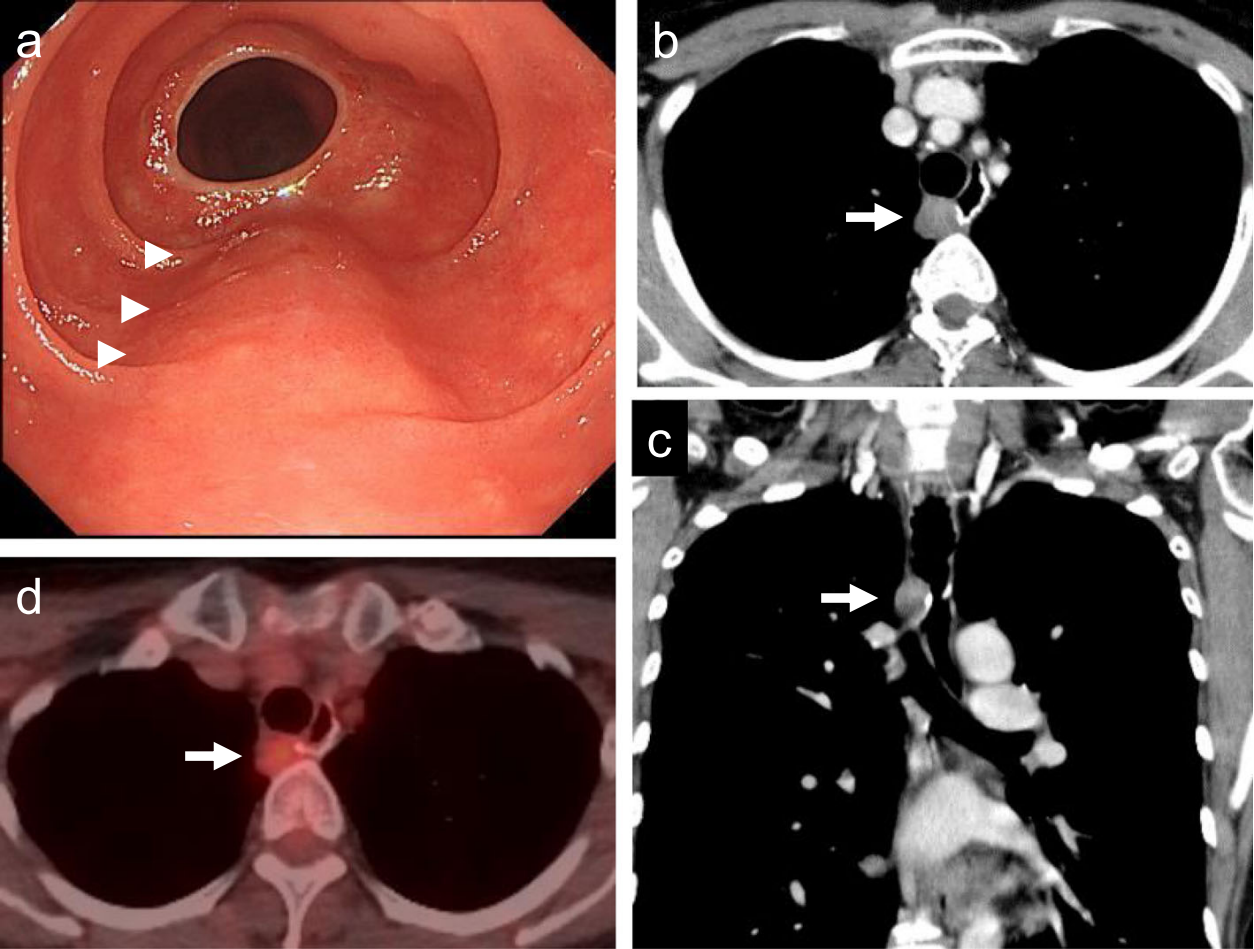
Images obtained before the third operation. Esophagoscopy showed a 20-mm-sized extrinsic mass located at the distal anastomotic site between the jejunum and the esophagus (arrowheads) (**a**). Contrast-enhanced CT showed a globular mass at the distal anastomotic site between the jejunum and the esophagus (arrow) (**b**, **c**). FDG-PET/CT revealed some FDG uptake in the tumor (arrow) (**d**)

**Fig. 4:**
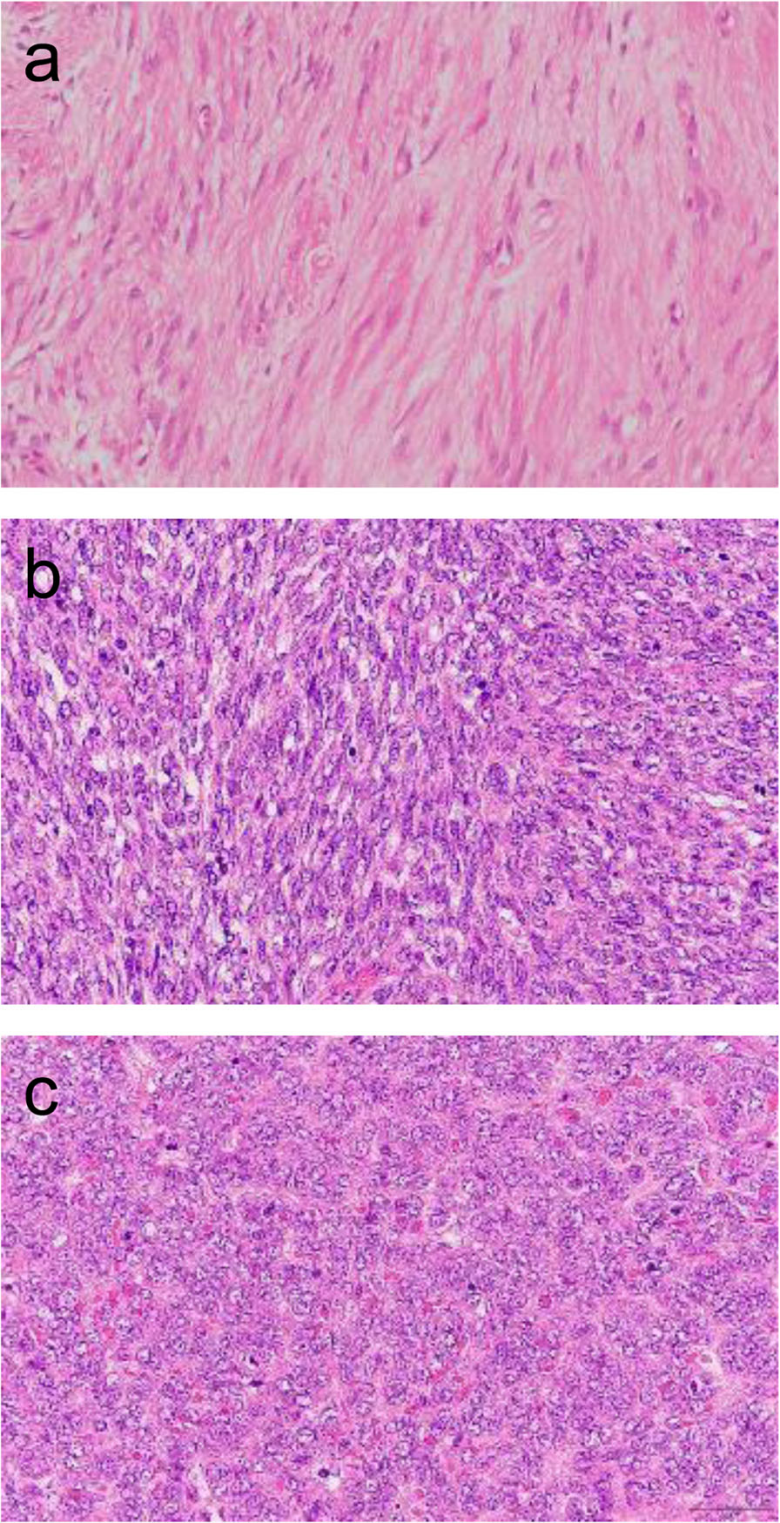
Photomicrographs of the tumors. The initial tumor (**a**). The secondary tumor (**b**). The tertiary tumor (**c**). The pathological grade was noted to increase for every surgery, based on the number of round cells, cellularity, the size of the nuclei, the degree of atypism, and the number of mitoses

Three years after the second surgery, SS recurred at the distal site of the anastomosis between the jejunum and the esophagus. Esophagoscopy showed a 20-mm-sized extrinsic mass (Fig. 3a). A contrast-enhanced CT revealed a globular mass (Fig. 3b, c), and FDG-PET/CT showed some FDG uptake in the tumor (Fig. 3d). The patient underwent thoracoscopic esophagectomy with gastric conduit reconstruction. Regarding malignancy, the pathological indicators of SS gradually became worse at every surgery based on the number of round cells, cellularity, the size of the nuclei, the degree of atypism, and the number of mitoses (Fig. 4a–c). Doxorubicin and ifosfamide constituted the first adjuvant therapy, which was then started and planned to be given for five courses. Three months after the surgery, she was free from tumor recurrence.

## Discussion

Primary esophageal SS is extremely rare, and only 14 cases of primary esophageal SS, including our case, have been reported to date. A summary of these cases is shown in Table 1 [5–17]. The age and sex distribution vary among the cases. The most common tumor location is the cervical to upper third of the thoracic esophagus. The gross type and histological type of all cases except for ours were the polypoid type and the biphasic type, respectively. The biphasic type consists of epithelial cells with spindle cells in various proportions, whereas the monophasic type consists of spindle cells alone and must be differentiated from other spindle cell tumors.

**Table 1:** A summary of the reported cases of primary synovial sarcoma of the esophagus

	Author, year, reference	Age	Sex	Location	Size (cm)	Gross type	Histological type	Translocation	Treatment	Follow-up
1	Palmer et al. 1983 [5]	75	F	Ut	2.5	Polypoid	Biphasic	–	S+RT	Dead, 24 m
2	Amr et al. 1984 [6]	25	M	Ut	5 × 3 × 1.5	Polypoid	Biphasic	–	S+RT	Alive, 36 m
3	Caldwell et al. 1991 [7]	29	F	Ut	–	Polypoid	–	–	S+RT	Alive, 195 m
4	Perch et al. 1991 [8]	15	M	Ce	10 × 6 × 3	Polypoid	Biphasic	–	S+RT	Alive, 78 m
5	Antón-Pacheco et al. 1996 [9]	14	F	Ce	7 × 6 × 6	Polypoid	Biphasic	–	S+CT+RT	Alive, 30 m
6	Habu et al. 1998 [10]	20	M	Ut	7.5 × 4 × 2	Polypoid	Biphasic	–	S+CT+RT	Alive, 20 m
7	Bonavina et al. 1998 [11]	63	F	Lt	5 × 5	Polypoid	Biphasic	–	S+CT	Alive, 13 m
8	Billings et al. 2000 [12]	47	M	EGJ	5.2 × 2.2 × 1.3	Polypoid	Biphasic	t(X;18)^a^	S	Alive, 21 m
9	Butori et al. 2006 [13]	72	F	UtMt	11.2 × 5.5	Polypoid	Biphasic	t(X;18)^a^	S+CT	Alive, 6 m
10	de Alencar et al. 2012 [14]	13	F	Ce	4 × 3	–	Biphasic	–	S	Alive, –
11	Niihara et al. 2015 [15]	70	M	Ce	7 × 3.3 × 1.4	Polypoid	Biphasic	t(X;18), SSX1	S, RT after recurrence	Dead, 42 m
12	Doroudinia et al. 2017 [16]	28	M	UtMt	10 × 10 × 5	Polypoid	Biphasic	–	S	–
13	Garcia-Rodriguez et al. 2019 [17]	54	F	Ut	7.8 × 4.6	SMT	Biphasic	t(X;18)^a^	CT+S	–
14	Present case	47	F	Ce	5.2 × 3.3 × 2	SMT	Monophasic	t(X;18), SSX2	S	Alive, 81 m

*F* female, *M* male, *Ut* upper thoracic esophagus, *Ce* cervical esophagus, *Lt* lower thoracic esophagus, *EGJ* esophagogastric junction, *Mt* middle thoracic esophagus, *SMT* submucosal tumor, *S* surgery, *RT* radiotherapy, *CT* chemotherapy, *m* months, *yr* years

^a^In their description, SS18-SSX fusion was positive. However, no further explanation was done

Although IHC for bcl-2, HHF35, and TLE-1 is useful for the diagnosis of spindle cell tumors, the diagnostic ability of IHC to detect SS is limited. Therefore, molecular analysis might be essential for diagnosing of SS. In at least 90% of the cases, the diagnosis of SS can be confirmed by detecting the chimeric transcript using RT-PCR. In rare instances, SS does not carry the *SS18*-*SSX* transcripts. These tumors may arise from alternative gene fusions (such as SS18L1/SSX1) or cryptic rearrangements. Since a novel SS18-SSX fusion-specific antibody was recently reported, this technology could provide high sensitivity and specificity for the diagnosis of SS [18]. To the best of our knowledge, the present case is the first report of primary esophageal SS presenting an SMT-like appearance and belonging to the monophasic type as confirmed by molecular detection of *SS18*-*SSX2* fusion transcripts. At least nine different *SSX* gene transcripts, *SSX1* to *SSX9*, have been identified, and the *SSX* gene transcript type is related to the histologic subtype and biological nature of the tumor. In SS, *SS18*-*SSX1* is the most common fusion subtype, followed by *SS18*-*SSX2*, and *SS18*-*SSX4* is very rare. *SS18*-*SSX1* tumors tend to belong to the biphasic subtype with a higher proliferative cell activity, entailing a higher risk of distant metastases. Meanwhile, *SS18-SSX2* tumors are more likely to be monophasic with a lower cell activity and with a more benign clinical course than other subtypes [19, 20].

Several factors indicative of a favorable outcome have been reported, including a patient age less than 25 years old, a tumor size less than 5 cm, and the absence of a poorly differentiated component [21].

With regard to treatment, a recent meta-analysis using one meta-analysis and four randomized controlled phase III trials including 2170 cases showed that adjuvant chemotherapy using doxorubicin ± ifosfamide was the best treatment option in terms of 5-year overall and disease-free survival in cases of soft tissue sarcoma [22]. For patients with localized SS, surgery with an adequate wide margin combined with adjuvant chemotherapy and/or radiation is regarded as the preferable treatment. Based on these data and considering the shortened periods until recurrence and the worsening of pathological indicators at every surgery, the first adjuvant therapy using doxorubicin and ifosfamide was started, with the plan of giving five courses after the tertiary surgery. For advanced patients with unresectable tumor and metastasis, doxorubicin and ifosfamide may be the front-line chemotherapy of choice. Furthermore, clinical trials showed effectiveness of high-dose ifosfamide alone [23], a combination of gemcitabine and docetaxel [24], pazopanib [25], and elibulin [26]. Trabectedin, a marine-derived antineoplastic drug and multitarget agent, which induces apoptosis and cell cycle arrest, is especially effective for translocation-related sarcoma, including SS [27, 28]. Phase 1 studies of the new potential therapies related to genetically engineered T lymphocytes and immunotherapeutic vaccines are ongoing.

## Conclusions

Monophasic SS, an extremely rare tumor in the esophagus, is difficult to distinguish from other spindle cell tumors. When an esophageal spindle cell tumor, especially of the polypoid type and located at the cervical to upper third of the thoracic esophagus, is observed, the possibility of SS should be considered. Molecular analysis appears useful for confirming the diagnosis of monophasic SS.

## Data Availability

Not applicable

## Abbreviations

SS: Synovial sarcoma
IHC: Immunohistochemistry
RT-PCR: Reverse transcription-polymerase chain reaction
SMT: Submucosal tumor
CT: Computed tomography
FDG-PET: ^18^F-fluorodeoxyglucose positron-emission tomography

## References

[1] Siegel HJ, Sessions W, Casillas MA, Said-Al-Naief N, Lander PH, Lopez-Ben R (2007). Synovial sarcoma: clinicopathologic features, treatment, and prognosis. Orthopedics..

[2] Eizinger F (1993). Soft Tissue Tumors. St. Louis.

[3] Haldar M, Hancock JD, Coffin CM, Lessnick SL, Capecchi MR (2007). A conditional mouse model of synovial sarcoma: insights into a myogenic origin. Cancer Cell..

[4] Clark J, Rocques PJ, Crew AJ, Gill S, Shipley J, Chan AM-L, et al. Identification of novel genes, SYT and SSX, involved in the t(X;18)(p11.2;q11.2) translocation found in human synovial sarcoma. Nat Genet. 1994;7:502–8. 10.1038/ng0894-502 7951320

[5] Palmer BV, Levene A, Shaw HJ (1983). Synovial sarcoma of the pharynx and oesophagus. J Laryngol Otol..

[6] Amr SS, Shihabi NK, Al HH (1984). Synovial sarcoma of the esophagus. Am J Otolaryngol..

[7] Caldwell CB, Bains MS, Burt M (1991). Unusual malignant neoplasms of the esophagus. Oat cell carcinoma, melanoma, and sarcoma. J Thorac Cardiovasc Surg..

[8] Perch SJ, Soffen EM, Whittington R, Brooks JJ (1991). Esophageal sarcomas. J Surg Oncol..

[9] Antón-Pacheco J, Cano I, Cuadros J, Vilariño A, Berchi F (1996). Synovial sarcoma of the esophagus. J Pediatr Surg..

[10] Habu S, Okamoto E, Toyosaka A, Nakai Y, Takeuchi M (1998). Synovial sarcoma of the esophagus: report of a case. Surg Today..

[11] Bonavina L, Fociani P, Asnaghi D, Ferrero S (1998). Synovial sarcoma of the esophagus simulating achalasia. Dis Esophagus..

[12] Billings SD, Meisner LF, Cummings OW, Tejada E (2000). Synovial sarcoma of the upper digestive tract: a report of two cases with demonstration of the X; 18 translocation by fluorescence in situ hybridization. Mod Pathol..

[13] Butori C, Hofman V, Attias R, Mouroux J, Pedeutour F, Hofman P (2006). Diagnosis of primary esophageal synovial sarcoma by demonstration of t (X; 18) translocation: a case report. Virchows Arch..

[14] de Alencar MH, Boldrini D, Costa Ade M, Torres de Oliveira AT, Attab CS (2012). Primary synovial sarcoma of the esophagus. Rev Col Bras Cir..

[15] Niihara M, Sato H, Kusafuka K, Kamiya S, Ashida R, Nakajima T (2015). Biphasic synovial sarcoma of the cervical esophagus confirmed by the presence of SYT-SSX1 fusion transcripts. Esophagus..

[16] Doroudinia A, Bakhshayesh Karam M, Dorudinia A, Mehrian P, Agha-Hosseini F (2017). Synovial sarcoma of the esophagus: a case report and review of literature. Middle East J Dig Dis..

[17] Garcia-Rodriguez V, Coronel E (2019). Esophageal synovial sarcoma diagnosed using EUS-guided fine-needle biopsy. Am J Gastroenterol..

[18] Baranov E, McBride MJ, Bellizzi AM, Ligon AH, Fletcher CDM, Kadoch C (2020). A novel SS18-SSX fusion-specific antibody for the diagnosis of synovial sarcoma. Am J Surg Pathol..

[19] Ladanyi M, Antonescu CR, Leung DH, Woodruff JM, Kawai A, Healey JH (2002). Impact of SYT-SSX fusion type on the clinical behavior of synovial sarcoma: a multi-institutional retrospective study of 243 patients. Cancer Res..

[20] Ren T, Lu Q, Guo W, Lou Z, Peng X, Jiao G (2013). The clinical implication of SS18-SSX fusion gene in synovial sarcoma. Br J Cancer..

[21] Garcia-Ortega D, Alvarez-Cano A, Martinez-Said H, Luna-Ortiz K, Caro-Sanchez C, Cuellar-Hubbe M (2019). Prognostic factors in synovial sarcoma. Experience of 173 cases in a high volume sarcoma center. Eur J Surg Oncol..

[22] O’Connor JM, Chacón M, Petracci FE, Chacón RD (2008). Adjuvant chemotherapy in soft tissue sarcoma (STS): a meta-analysis of published data. J Clin Oncol..

[23] Lee SH, Chang MH, Baek KK, Han B, Lim T, Lee J (2011). High-dose ifosfamide as second- or third-line chemotherapy in refractory bone and soft tissue sarcoma patients. Oncology..

[24] Maki RG, Wathen JK, Patel SR, Priebat DA, Okuno SH, Samuels B (2007). Randomized phase II study of gemcitabine and docetaxel compared with gemcitabine alone in patients with metastatic soft tissue sarcomas: results of sarcoma alliance for research through collaboration study 002 [corrected]. J Clin Oncol..

[25] Kasper B, Sleijfer S, Litière S, Marreaud S, Verweij J, Hodge RA (2014). Long-term responders and survivors on pazopanib for advanced soft tissue sarcomas: subanalysis of two European Organisation for Research and Treatment of Cancer (EORTC) clinical trials 62043 and 62072. Ann Oncol..

[26] Schöffski P, Chawla S, Maki RG, Italiano A, Gelderblom H, Choy E (2016). Eribulin versus dacarbazine in previously treated patients with advanced liposarcoma or leiomyosarcoma: a randomised, open-label, multicentre, phase 3 trial. Lancet..

[27] Demetri GD, Von Mehren M, Jones RL, Hensley ML, Schuetze SM, Staddon A (2016). Efficacy and safety of trabectedin or dacarbazine for metastatic liposarcoma or leiomyosarcoma after failure of conventional chemotherapy: results of a phase III randomized multicenter clinical trial. J Clin Oncol..

[28] Kobayashi H, Iwata S, Wakamatsu T, Hayakawa K, Yonemoto T, Wasa J (2020). Efficacy and safety of trabectedin for patients with unresectable and relapsed soft-tissue sarcoma in Japan: a Japanese Musculoskeletal Oncology Group study. Cancer..

